# Erectile function outcomes following surgical treatment of ischemic priapism

**DOI:** 10.1016/j.amsu.2022.103696

**Published:** 2022-04-29

**Authors:** Moez Rahoui, Yassine Ouanes, Chaker Kays, Bibi Mokhtar, Kheireddine Mrad Dali, Ahmed Sellami, Sami Ben Rhouma, Yassine Nouira

**Affiliations:** Urology Department La Rabta Hospital, Tunis, Tunisia

**Keywords:** Priapism, Erectile dysfunction, Sexuality, Prognosis, Surgery

## Abstract

**Introduction:**

Ischemic Priapism is defined as an abnormally prolonged state of erection, exceeding 6 h, often and irreducible, occurring without any sexual stimulation. Ischemic priapism has a fatal consequence on the sexual function of men if it's not promptly managed. This pathology can cause erectile dysfunction and this can alter the quality of life of patients.

**Objective:**

The aim of our study was to determine the factors influencing erectile function after treatment of ischemic priapism.

**Patients and methods:**

This is a ten-year retrospective, descriptive and analytic study of 40 patients who consulted the urology department at the university hospital center for treatment of ischemic priapism (2010–2019).

**Results:**

We included 40 patients in our study. The mean age was 35.2 [18–62]. Duration of priapism varied from 20 to 360 h (mean 76.6). The most common etiology of priapism was sickle cell disease in 65% of cases. The mean preoperative IIEF-5 score was 23 [21–26]. All patients underwent corporal aspiration with an injection of ephedrine, but detumescence was observed in only 10% of cases. Thirty-six patients had a distal shunt with detumescence in approximately 70% of cases. Eleven patients underwent a distal shunt but seven patients had definitive fibrosis. After the episode of priapism, only eight patients retained normal erectile function. The mean postoperative IIEF-5 score was 14 [ 7−26]. We noted an improvement in erectile function in 8 patients treated with tadalafil. In multivariate analysis, we have demonstrated that a treatment delay exceeding 48 h, fibrosis and the necessity of a distal shunt significantly affects postoperative erectile function (p = 0.001; p = 0.002; p = 0.002 respectively).

**Conclusion:**

According to our study, delayed management exceeding 48 h, fibrosis and the necessity of a surgical distal shunt are three independent factors affecting erectile function after treatment of ischemic priapism.

## Highlights

• Ischemic Priapism is defined as an abnormally prolonged state of erection.
• This is a real medical and surgical emergency.
• Ischemic priapism has a fatal consequence on the sexual function of men if it's not promptly managed.
• The treatment delay exceeding 48 h, fibrosis and the necessity of a distal shunt significantly affects postoperative erectile function.

## 1 Introduction

Ischemic Priapism is defined as an abnormally prolonged state of erection, exceeding 6 h, often and irreducible, occurring without any sexual stimulation [1]. This is a real medical and surgical emergency because only the precocity of its treatment, gives chances of preventing erectile dysfunction by fibrosis of the corpora cavernosa. Treatment should be initiated as quickly as possible to prevent irreversible complications, including erectile dysfunction. The main objective of the treatment of ischemic priapism is to decrease cavernous pressure and increase arterial flow to restore satisfactory oxygenation of the cavernous tissue. Currently, aspiration and irrigation with a vasoactive agent is the first-line treatment for IP. Bypass surgery should be considered as a second line treatment [2]. The therapeutic results remain disparate according to the authors. However, all authors agree underline the poor prognosis of the condition with more than 50% of erectile dysfunction in often young patients with severe social and psychological consequences. The aim of our study was to determine the factors influencing erectile function after surgical treatment of ischemic priapism. This work has been reported according to SCARE 2020 criteria [3].

## 2 Patients and Methods

It was a retrospective, observational study conducted in a tertiary care center. After gaining local ethics committee approval, we included all the patients who were presented with first episode of ischemic priapism to our department from January 2000 to December 2020. The diagnosis of ischemic priapism was based on the clinical context and the realization of the blood gas of the corpus cavernosum. Patients with recurrent or non-ischemic priapism were not included. A total of forty patients were studied. On admission, age, detailed history of duration of the priapism, presence of penile pain, drug intake, local or perineal trauma, hematological diseases, neurologic conditions, and erectile dysfunction (ED) were taken. Treatment begins with analgesic treatment and hyperhydration. Our protocol was based on an initial medical treatment which consisted of a paracetamol-based analgesic treatment, oral and intravenous hydration (2 L/24H) associated with an injection (IIC) of an alpha-stimulant. The technique consists of the injection of 0.5 mg of phenylephrine, to be repeated every 10–15 min–15 min until detumescence is obtained, diluted in 2 cm^3^ of physiological serum directly by lateral puncture of the penis in a cavernous body. In case of persistence of the erection, this procedure can be repeated every 10–15 min until detumescence is obtained, but not exceeding 3 mg of alpha-stimulant. In case of failure of medical treatment, corporal aspiration and irrigation were performed. If penis failed to detumescent after giving 1-h of conservative management, we performed open distal shunt under general anesthesia according to the Al Ghorab technique. The success of the treatment was based on obtaining a total detumescence. Fibrosis was diagnosed clinically. Patients with erectile dysfunction were treated with Tadalafil 5 mg daily. Erectile function was evaluated with International Index of Erectile Function-5 questionnaire on admission and during follow-up (three month and six months). All factors that could potentially influence erectile function were analyzed using SPSS version 20. The statistical analysis was carried out using Chi-squared test and a logistic regression.

## 3 Results

A total of 40 patients were managed for ischemic priapism in our department with a mean age of 35.2 years (range 18–62). The presentation delay varied from 20 h to 360 h (mean = 76.6 h) and almost of s (62%) presented 36 h after the onset of priapism. The diagnosis was retained by performing a blood gas of the corpora cavernosa confirming the ischemic nature of priapism. The most common etiology of priapism was sickle cell disease in 65% of cases. By history, all patients had no problems with erectile function before penile fracture, only two patients had risk factors for systemic vascular diseases at first presentation, such as diabetes mellitus (one patient) and hypertension (one patient). The mean preoperative IIEF-5 score was 23 [21–26]. In our series, all patients received medical treatment which is based on an analgesic, a hydration and an injection of ephedrine. Failure of medical treatment was noted in all cases. All patients underwent corporal aspiration with an injection of ephedrine. Immediate success was noted in only 10% patients. 36 patients underwent surgical treatment either 90% of cases. The surgery was performed under general anesthesia in 24 patients and under spinal anesthesia in 12 patients s. All patients had a distal spongio-cavernous shunt according to Al-Ghorab. Detumescence was observed in approximately 70% of cases. Seven patients underwent a distal shunt but had definitive fibrosis. There was no significant postoperative morbidity except for two patients who had mild wound infection. After 06 months of follow-up, the mean postoperative IIEF-5 score was 14 ± 4.After the episode of priapism, only eight patients retained normal erectile function. We treated patients who complained of erectile dysfunction during follow-up with tadalafil 5 mg daily. With treatment with tadalafil, only eight patients had an improvement in their erectile function. The remaining 24 patients showed no improvement in erectile function with tadalafil during follow-up.). Three of the four patients (75%) treated with aspiration and ICI/aspiration alone had normal erectile function after treatment. Preservation of normal erectile function after distal bypass procedures is 13.88%. After treatment with tadalafil, the mean postoperative IIEF-5 score was 17 [14–20]. (Table 1).

**Table 1:** Demographics and descriptive analysis.

Mean age (range)	35.2 years (16; 62)
**Causes of ischemic priapism n (%)**	
Sickle cell disease	26 (65)
Malignant hemopathy	4 (10)
Neuroleptic drugs	3 (7.5)
Sildenafil	3 (7.5)
Idiopathic	4 (10)
Presentation delay	76 h
Preoperative erectile function	21 ± 3
**Management n (%)**	
Medical treatment	40 (100)
Aspiration + Ephedrine	40 (100)
Distal shunt	36 (90)
**Erectile function 6 months after surgery**	
Mean IIEF-5 score	14 ± 4.
Erectile dysfunction rate n (%)	32 (80)
Fibrosis (%)	7 (17.5)
**Erectile function after tadalafil treatment**	
Mean IIEF-5 score	17 ± 3
Erectile dysfunction rate n (%)	24 (60)

On multivariate analysis, we found that a treatment delay exceeding 48 h, fibrosis and the necessity of a distal shunt significantly affects postoperative erectile function (p = 0.001; p = 0.002; p = 0.002 respectively) Table 2.

**Table 2:** Relationship between factors related to the patient and the intervention, and sexual function.

	Erectile dysfunction
Age	*P* > 0.05
Sickle cell disease	*P* > 0.05
Delay >48 h	P = 0.001
Medical treatment	*P* > 0.5
Aspiration	*P* > 0.05
Fibrosis	P = 0,002
Distal shunt	*P* = 0.002

## 4 Discussion

Priapism is characterized by a prolonged penile erection in the absence of sexual interest or desire. It is a urological emergency because it can damage erectile tissue and can lead to erectile dysfunction [2]. Priapism affects all age groups. The incidence is as high as 3.6% in adolescents (<18 years) and increases to 42% in adult patients [4]. Priapism is classified into two types according to the degree of oxygenation of the blood in the corpora cavernosa, low-flow (ischemic) and high-flow (non-ischemic) priapism [5]. Originally described in 1934, ischemic priapism represents the most common form and can occur present as a recurrent event or episodes [6,7]. Ischemic priapism, also called veno-occlusive priapism or low flow priapism, is a persistent erection marked by rigidity of the corpora cavernosa in the absence of an arterial cavernous inflow [[7], [8]]. It consists of an imbalance of vasoregulatory mechanisms, predisposing the penis to an ischemic environment. Priapism is most often associated with hematological pathologies, such as glucose-6-phosphate dehydrogenase deficiency, sickle cell anemia, thrombophilia and hyperviscous states. It may be secondary to the use of certain drugs such as neuroleptics, sildenafil, selective serotonin reuptake inhibitors [9]. Other causes have been identified such as cancers (most commonly bladder, prostate, kidney and colorectal), total parenteral nutrition, drug use such as cocaine, severe alcohol abuse and trauma [10]. Understanding the risk factors for priapism commonly associated disease states in the clinic to establish an accurate and rapid diagnosis. Prompt diagnosis of the type of priapism is essential because ischemic priapism requires immediate treatment to maintain good sexual function. Doppler ultrasound and body aspirate blood gas analysis have been recommended to differentiate the type of priapism [11]. In our series, the History, the clinical context and blood gas from the corpus cavernosum made it possible to identify the ischemic origin of priapism. The initial treatment of ischemic priapism, based on immediate penile aspiration, should be initiated without delay whatever the cause [12]. In our study, aspiration with intracavernous injection of phenylephrine resulted in complete detumescence in four patients. The overall success rate was 10%. The reported success rate of aspiration alone is around 30% and intracavernous injection with or without aspiration ranges from 43% to 81% [13]. In our series, the lower success rate was probably due to a presentation delay >48 h. Open distal shunts like the Al-Ghorab technique that was used in our series were successful in obtaining detumescence in 28 out of 36 patients (70% success rate). In the literature, The Al-Ghorab shunt has a success rate of approximately 74% [14]. Faced with the failure to obtain detumescence after a distal shunt, a second shunt was performed, but definitive fibrosis was observed in 7 patients. The assessment of sexual function was based on the use of the IIEF-5 questionnaire in patients treated before the priapism event and during the 6-month follow-up, which is a validated tool [15,16]. Of 34 sexually active men, only 8 patients retained normal erectile function. This poor result was due to the prolonged duration of priapism (without ED the mean was 76.6 h, while patients with the mean ED were 108.6 h). Zheng et al. in their series reported poor erectile function after treatment for priapism (severe ED present in six out of eight patients) with an average duration of priapism of 96.33 h [17]. Gottsch and al. reported an adequate erection in only six of 35 patients after treatment for priapism [18]. Tabibi and al. reported normal erectile function in 33.3 of patients after an Al-Ghorab shunt. The mean duration of priapism was 51.12 h [19]. Our result also shows poor preservation of erectile function after Shunt surgery. In our study, the preservation of normal erectile function after the distal shunt was 13.8%. We discovered in our series that presentation time delay >48, fibrosis and the necessity of a distal shunt significantly affects postoperative erectile function (p = 0.001; p = 0.002; p = 0.002 respectively). The use of tadalafil improved erectile function in 8 patients (40%) in our series. Kumar and all reported improvement of erectile dysfunction in two out of 14 patients treated with tadalafil [20]. Apart from improving erectile function, some studies have suggested that the use of IPDE 5 may prevent recurrence of priapism. These studies suggest that daily administration of low-dose PDE-5 inhibitors after the acute period of priapism to upregulate PDE-5 gene expression, stimulate eNOS expression, and reduce the state of the dysfunctional NO pathway [21]. In doing so, such treatment could restore the balance between stimulating and inhibiting factors, reducing episodes of priapism [22]. In a small series of cases, Burnett and colleagues [23] have shown that daily treatment with a PDE5 inhibitor reduced episodes of ischemic priapism, without modifying erectile function. Hence, we concluded that the follow up should be extended to reach a more definitive conclusion on the degree of erectile dysfunction.

The small sample size and retrospective nature of our study were the main limitations.

## 5 Conclusion

Priapism is a major andrological emergency and must be treated immediately. The prognosis depends mainly on the age of the patients and the time of their care. Ischemic priapism has a devastating consequence on the sexual function of men if it's not promptly managed. According to our study, the treatment delay exceeding 48 h, fibrosis and the necessity of a distal shunt significantly affects postoperative erectile function.

## Funding

We have any financial sources for our research.

## Provenance and peer review

Not commissioned, externally peer-reviewed.

## Ethical approval

Not applicable.

## Sources of funding

This research did not receive any specific grant from funding agencies in the public, commercial, or not-for-profit sectors.

## Author statement

Rahoui Moez, Yassine ouannes and Kais chaker: Data collection, Manuscript writing, Results discussion.

Bibi Mokhtar, Kheireddine Mourad daly and Ahmed sellami: Manuscript writing and revision.

Ben rhouma sami and Nouira yassine: Paper revision.

## Registration of research studies

1. Name of the registry: N/a
2. Unique identifying number or registration ID: N/a
3. Hyperlink to your specific registration (must be publicly accessible and will be checked): N/a

## Guarantor

Rahoui Moez is the guarantor of the study and accept full responsibility for the work and/or the conduct of the study, had access to the data and controlled the decision to publish.

## Consent

Written informed consent was obtained from the patient for publication of this case report and accompanying images. A copy of the written consent is available for review by the Editor-in-Chief of this journal on request.

## Declaration of competing interest

The authors declare that they have no known competing financial interests or personal relationships that could have appeared to influence the work reported in this paper.
